# Sports Bras Improve Chest Keloids but Outcomes Are Dependent on Breast Size: A Retrospective Analysis

**DOI:** 10.3389/fonc.2022.871115

**Published:** 2022-07-08

**Authors:** Yanting Zhu, Qiguo Zhang, Ting Gong, Peng Zhang, Bo Cheng, Jian Liu, Chao Ji

**Affiliations:** ^1^ Department of Dermatology, The First Affiliated Hospital of Fujian Medical University, Fuzhou, China; ^2^ Key Laboratory of Skin Cancer of Fujian Higher Education Institutions, The First Affiliated Hospital, Fujian Medical University, Fuzhou, China; ^3^ Fujian Dermatology and Venereology Research Institute, The First Affiliated Hospital, Fujian Medical University, Fuzhou, China; ^4^ The Department of Dermatology, The First Affiliated Hospital of Xiamen University, Xiamen, China; ^5^ Central Laboratory, The First Affiliated Hospital of Fujian Medical University, Fuzhou, China; ^6^ Department of Dermatology MINE BUTY, Fuzhou, China

**Keywords:** keloid, breast size, sports bra, skin tension, postoperative management, treatment outcome

## Abstract

Our study is a retrospective medical record review performed on 95 female keloid patients with the standard therapy combining complete surgical excision with superficial X-ray radiation. We aimed to analyze the relationship between breast size and treatment outcomes as well as the benefits of sports bras in the postoperative management of keloids. The results showed that the keloid score of no sports bra group was significantly worse than the score of sports bra group at 1-year follow-up. In addition, the large breast size group showed more significant improvement of keloid score when wearing sports bras. Our study highlights that continuous wearing a sports bra effectively reduces the skin tension of the postoperative incision and promotes recovery, especially for patients with large breast size.

## Introduction

Keloids are benign skin tumors in which raised scar tissue grows excessively and invasively beyond the original wound margins (1). They occur more frequently at certain body sites with highly mobility and high tension in susceptible individuals. The anterior chest wall is one of the most common keloid-prone sites (2). It was previously thought that the periodic stretching force produced by the daily movement of the upper arm was a factor in the formation of high tension on the skin of anterior chest (3). However, little attention has been paid to the effect of gravity-induced skin strain of breast on the tension of anterior chest skin.

Our surgeon observed the phenomenon that patients with larger breasts did not respond well to keloid treatments and had higher recurrence rates than those with smaller breasts. Then, we began to recommend patients to wear high-supportive sports bras after operation indiscriminately. We recommended our patients to wear the same kind of sports bras in different sizes to accommodate different breast sizes. The surgeon noted the dimensions of breasts for each patient in their medical records. However, not all patients followed doctor’s advice due to some personal reasons. Later, we found that sports bras did bring an improved outcome for those patients who persistently wore the sports bras. Therefore, we conducted this retrospective medical record review which included 95 female keloid patients with a standard combination of both surgical excision and superficial X-ray radiation therapy in MINE BUTY Hospital between January 2018 and July 2020. We aimed to analyze the relationship between breast size and treatment outcomes as well as the benefits of sports bras in the postoperative management of keloids.

## Patients and Methods

All patients were clinically diagnosed with anterior-chest keloid (keloids located above the upper border of the breast). JSW Scar Scale (JSS) 2015 were used for assessing the keloid scores at 1-year follow-up (4). Breast size score (BSS) (0–18) were converted from under-bust and over bust measures using a system conceptually similar to sizing unilateral breast prostheses (5). To minimize the selection bias, patients with complete medical history were randomly chosen for our retrospective study, matching in age, time of onset, keloid size, vertical width of keloid, previous treatment and breast size score. All participants were treated with surgical resection followed by post-operative X-ray radiation therapy using Sensus SRT- 100 irradiator. Every patient received a total dose of 15 Gy at 50KV X-ray in 4 fractions within one month after operation. To minimize the potential risk of adverse effects of radiation, all patients in our study received the first 6Gy of radiotherapy immediately after the operation. The remaining 9Gy was completed in the following three weeks (3Gy per week). Radiation was delivered with the area around the wound protected by 0.763mm thick lead shield to avoid any unnecessary radiation exposure. Among these patients, 50 patients wore recommended sports bras 20 hours/day for 6 months after surgery (sports bra group) while the other 45 patients didn’t wear any bras after surgery (no sports bra group). Descriptive statistics including mean, standard deviation, median (25th percentile-75th percentile), or crude numbers (percentage) were used to describe the demographics and disease characteristics. A comparison was made between the two groups using the Student t-test and Manne Whitney tests. Linear regressions were performed and a standardized Kendall‘ s tau- b coefficient was reported. Statistical analyses were conducted with GraphPad Prism 9 for Windows. *P*<0.05 was considered statistically significant.

## Results

Baseline demographics and disease characteristics were shown in **Table 1**. The keloid scores in no sports bra group (median=3) were significantly worse than the score of sports bra group (median=1) at 1 year following up. The clinical pictures of patients in both groups before and one year after operation were showed in **Figure 1**. The patients in sports bra group exhibited milder erythema and telangiectasia and smaller vertical width of lesion one year after treatment compared to patients in no sports bra group. To further inspect the differential keloid scores among different breast sizes, patients were grouped into large breast (BSS>7.7) and small group (BSS<7.7) (6). The large breast size group showed more significant improvement of keloid scores when patient wore sports bras than the small breast size group (P=0. 0058). Patient self-assessment questionnaire at 1 year following up showed similar result to the keloid score (**Table 1**). Larger size of the breast was correlated to the worse keloid score in both groups. However, the positive association in no sports bras group was stronger compared to sports bras group. Therefore, it was suggested that sports bras were more important for patients with large breasts in improving the outcome of treatment.

**Table 1:** Demographic and clinical characteristics of female patients with prothoracic keloid.

Characteristics	Patients in no sports bra group(n = 45, 47%)	Patients in sports bra group(n = 50, 53%)	*P* value
Age, y, mean (SD)	34.02(11.66)	35 (13.91)	.713
Time of onset, y, mean (SD)	6.84 (4.43)	6.86 (5.43)	.9879
Keloid size, cm², mean (SD)	23.54 (21.95)	16.5(17.98)	.3449
≥20cm², n (%)	31 (0.69)	42 (0.84)	0.0936
<20cm², n (%)	14 (0.31)	8 (0.16)	
Vertical width, mm, mean (SD)	4.333	3.860	0.0918
Times of previous treatment, n, mean (SD)	3.689	4.120	0.4742
Intralesional corticosteroids	2.533	2.780	0.5688
Other treatment	1.156	1.340	0.6302
#Classification score, mean (SD)	18.96 (2.39)	19.31 (1.97)	.4349
Breast size score, mean (SD)	6.91 (1.99)	6.64 (1.89)	.4976
>7.7, n (%)	27 (0.6)	34 (0.68)	0.5210
<7.7, n (%)	18 (0.4)	16 (0.32)	
#Keloid score at 1 year following up, median (IQR)	3 (2-3)	1 (1-2)	<.0001 ****
Breast size score<7.7, median (IQR)	2 (1-2.5)	1 (1-1)	.0058**
Breast size score>7.7, median (IQR)	3 (3-4)	2 (2-2)	<.0001 ****
#Patient self-assessment questionnaire at 1 year following up	2 (2-2)	1 (1-1)	<.0001 ****
Breast size score<7.7, median (IQR)	2 (1-2)	1 (1-1)	<.0001 ****
Breast size score>7.7, median (IQR)	2 (2-2)	1 (1-1)	<.0001 ****

#Classification score and keloid score (evaluation score) were quantified using Japan Scar Workshop Scar Scale 2015. Patient self-assessment questionnaire were designed based on Global Assessment of Improvement from Baseline. Breast size score (BSS) (0–18) were converted from under-bust and over bust measures using a system conceptually similar to sizing unilateral breast prostheses. SD, standard deviation; IRQ, interquartile range. P**≤0.01, P****≤0.0001.

**Figure 1:**
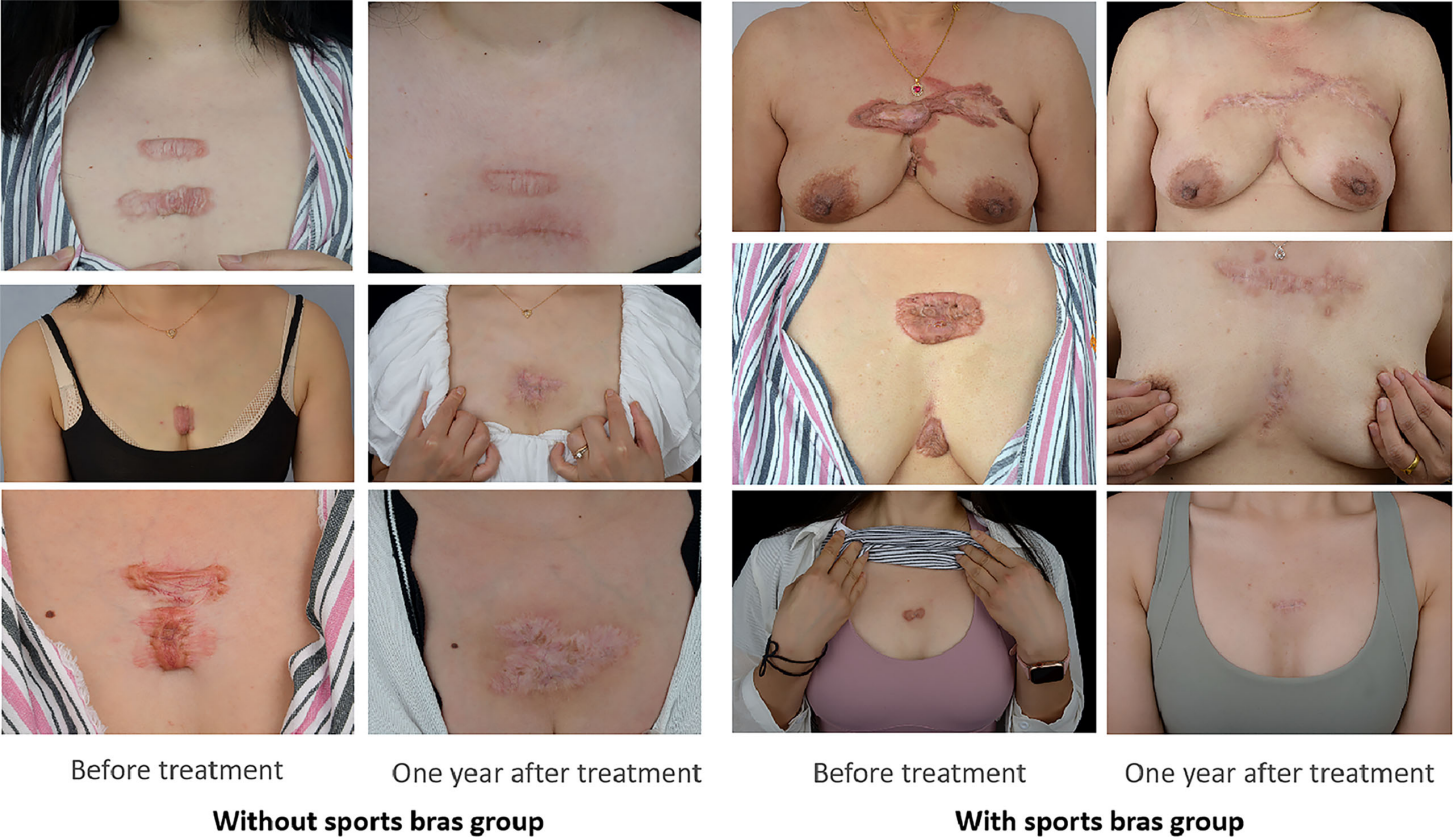
The clinical pictures of patients in both groups before and one year after operation. The patients in sports bra group exhibit milder erythema and telangiectasia and smaller vertical width of lesion one year after treatment compared to patients in no sports bra group.

## Discussion

**Figure 2:**
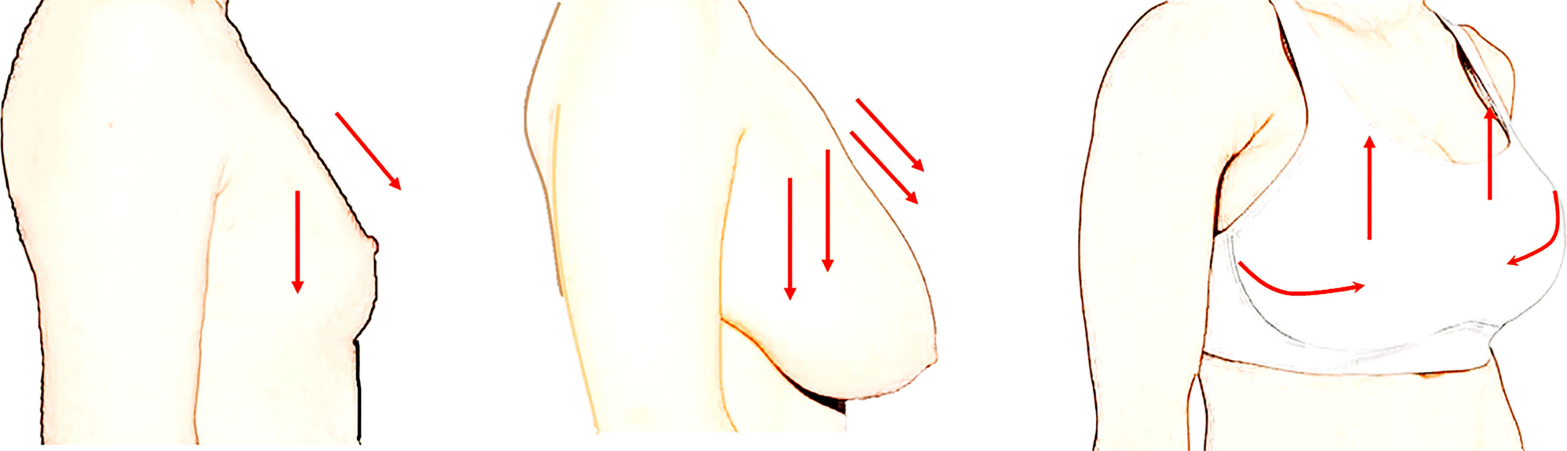
Schematic drawing of skin tension induced by breast and the effect of sports bras. Breast size was considered to be an important factor inducing high skin strain on anterior-chest. Wearing sports bras after surgery can reduce ongoing tension and thus consistently improve keloid management and prevent keloid recurrence.

Keloid is a complex disease and the pathogenesis has not been clarified. Many factors are associated with its recurrence and progression, such as infection, recurrence of the primary lesion, surgical approach, postoperative care, location and skin tension (7). In this study, we focused on the influence of prothoracic skin tension on keloid treatment. Scurr Amy Sanchez’s work (8) showed that potentially damaging static prothoracic skin strains (up to 75% peak strain) was caused by gravitational loading. Particularly high skin strains were observed longitudinally in the upper-outer breast region for larger breast women. We have tried to analyze the relationship between BSS and keloid size at beginning of the study. However, we didn’t find significant difference between BSS and keloid size due to small sample size and recall bias. We think we can provide an alternative explanation to the association between BSS and keloid size *via* comparing keloid improvement and BSS. Our study highlights that maintaining wearing a sports bra is effective in reducing the ongoing chest skin tension of the postoperative incision and achieving satisfactory results, especially for patients with large breast size. These concepts were schematically illustrated in **Figure 2**.

Keloids have suboptimal treatment outcomes, with recurrence rates of up to 100% with surgical treatment alone and 50% with medical management (9, 10). Currently, post-surgical adjunctive radiation therapy is one of the most effective methods for recurrent keloids, but its recurrence rate is still approximately 20% (11). Thus, increasing researches on reducing recurrence rate of keloid have been reported. Wearing supported sports bras may be an alternative in combination with the other traditional keloid treatment methods for optimal treatment outcomes. More education about breast management of tension reducing is required for keloid patients.

Due to the limitations of retrospective nature and small sample size, selection and unmeasured confounding bias cannot be completely excluded inevitably. Further controlled prospective study at the multi-center level is necessary to evaluate the results.

## Data Availability Statement

The datasets presented in this study can be found in online repositories. The names of the repository/repositories and accession number(s) can be found below: https://data.mendeley.com/datasets/gwyfc7bnzb/3.

## Ethics Statement

Written informed consent was obtained from the individual(s) for the publication of any potentially identifiable images or data included in this article.

## Author Contributions

YZ, QZ and TG contributed equally to this article. YZ, TG, JL and CJ donceptualized and designed the study; YZ, QZ and PZ wrote the manuscript; GT, BC and CJ revised the article critically for important intellectual content. YZ, PZ and JL collected clinical pictures and analyzed data. All authors contributed to the article and approved the submitted version.

## Funding

The Natural Science Foundation of Fujian Province (2020J02053), Fujian Provincial Health Commission (2018-ZQN-83) and Fujian Provincial Department of Science and Technology(2021J011341).

## Conflict of Interest

The authors declare that the research was conducted in the absence of any commercial or financial relationships that could be construed as a potential conflict of interest.

## Publisher’s Note

All claims expressed in this article are solely those of the authors and do not necessarily represent those of their affiliated organizations, or those of the publisher, the editors and the reviewers. Any product that may be evaluated in this article, or claim that may be made by its manufacturer, is not guaranteed or endorsed by the publisher.
